# Hydrogen‐bond‐mediated retention of a phenolic SNAP analogue in polyvinyl alcohol/PEG hydrogels for sustained nitric oxide release and antibacterial activity

**DOI:** 10.1002/smo2.70099

**Published:** 2026-08-31

**Authors:** Yixin Zhu, Peixuan Wu, Shuting Zhang, Congjun Xu, Guanghua Xia, Jiangli Fan, Nicola E. Brasch, Yang Zhou, Yuanyuan Liu

**Affiliations:** ^1^ Key Laboratory of Advanced Materials of Tropical Island Resources of Ministry of Education and School of Chemistry and Chemical Engineering Hainan University Haikou China; ^2^ Key Laboratory of Tropical Biological Resources of Ministry of Education and One Health Institute School of Pharmaceutical Sciences Hainan University Haikou China; ^3^ State Key Laboratory of Fine Chemicals Dalian University of Technology Dalian China; ^4^ School of Science Auckland University of Technology Auckland New Zealand

**Keywords:** antibacterial agent, hydrogel, hydrogen bonding, nitric oxide

## Abstract

Nitric oxide (NO)‐releasing materials have attracted considerable interest for their potential in diverse biomedical applications. However, a persistent challenge is the leaching of NO donors from these materials, which leads to significantly reduced NO release durations and undesirable burst release effects. Herein, we demonstrate a novel hydrogen‐bonding strategy to address NO donor leaching and the burst release of NO. A nitrosothiol‐based NO hydrogel was developed by incorporating a SNAP analog bearing a 4‐hydroxyphenethyl sidechain into polyvinyl alcohol (PVA)/PEG hydrogels. The hydrogel containing the structurally modified SNAP analog exhibited an elongation of over 300% and sustained NO release under physiological conditions. Hydrogen‐bonding interactions contributed to the enhanced retention of the SNAP analog within the hydrogel network, extending donor retention to 144 h compared with the hydrogel containing unmodified SNAP (10 h). These NO‐releasing hydrogels exhibited potent antibacterial activity against both *S*. *aureus* and *E*. *coli*, with bacterial reduction exceeding 99%, and caused pronounced disruption of surface‐associated bacterial structures. This novel hydrogen‐bonded NO donor–hydrogel system offers a promising strategy for durable and controlled NO release, with potential applications in wound dressings, tissue scaffolds, and flexible sensors.

## Graphical abstract

A hydrogen‐bond‐donating NO donor was designed to interact specifically with the hydrogel network, thereby minimizing donor leakage and achieving prolonged NO release.

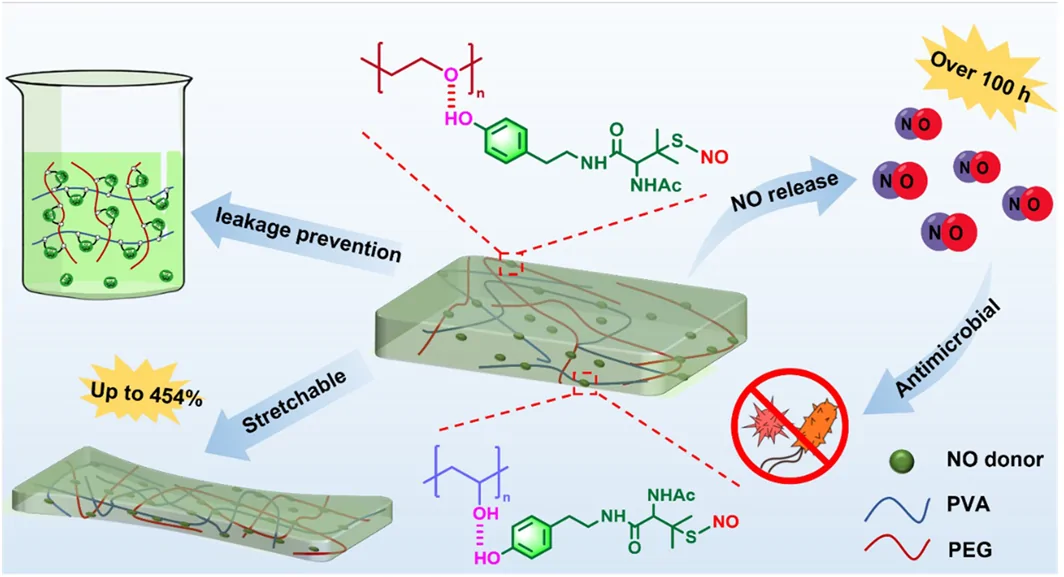

## 1 INTRODUCTION

Nitric oxide (NO), an endogenous diatomic radical gas, serves as a critical signaling molecule and effector, regulating diverse physiological processes in organisms.[^1^, ^2^, ^3^] Within the human body, it modulates vasodilation, participates in neuronal retrograde signaling, and exerts key functions in inflammation and wound healing, as well as having antibacterial, antimicrobial, and antitumor properties.[^4^, ^5^, ^6^] However, the gaseous nature of NO presents substantial challenges for its clinical application, including difficulties in controlling its release and risks associated with its inherent toxicity at elevated concentrations.[^7^, ^8^] Addressing these limitations requires strategies that enable precise, localized delivery of NO at therapeutic dosages.^[9]^ Consequently, the development of exogenous donor molecules and materials capable of controlled NO release is essential for advancing its therapeutic and biomedical utility.[^10^, ^11^, ^12^]

Current research on the development of controllable NO‐releasing biomedical devices focuses on polymer‐based materials, functionalized either by chemical grafting of NO‐donor groups or physical doping with NO donors.[^13^, ^14^] These strategies seek to emulate the endogenous, sustained NO release from the vascular endothelium under physiological conditions.[^15^, ^16^] Although the doping approach enables prolonged NO release lasting from days to months and surpasses certain grafting methods in the duration of NO release,^[17]^ it is substantially limited by donor leaching. This process depletes the NO reservoir, shortens release longevity, and triggers uncontrolled local NO bursts that can provoke oxidative stress and cytotoxicity.^[18]^ For example, *S*‐nitroso‐*N*‐acetylpenicillamine (SNAP)‐doped silicone rubber and a SNAP‐incorporated polyurethane catheter demonstrated SNAP leaching of 11% (7 d) and 47% (22 d), respectively.^[19]^ Suppressing NO donor leaching is essential to achieve stable and biocompatible NO‐releasing polymer systems.

Anti‐leakage strategies for embedded NO donors in polymer‐based systems remain limited. To address donor leaching, Meyerhoff et al. utilized fluorine‐fluorine interactions between a fluorinated *S*‐nitrosothiol and a PVDF‐HFP polymer matrix, achieving minimal leaching of only 0.6% over 9 d.^[20]^ Separately, the Handa group incorporated silicone oil loaded with SNAP into silicone rubber, which reduced donor leakage by 45% within 24 h compared to the non‐oil formulation.^[21]^ However, these strategies lack generalizability and cannot be efficiently extended to a wide range of polymers used in biomedical applications. Therefore, developing other scalable methods for controlling donor leakage is essential to enable the broader application of durable and efficient NO‐releasing polymer systems.

**FIGURE 1:**
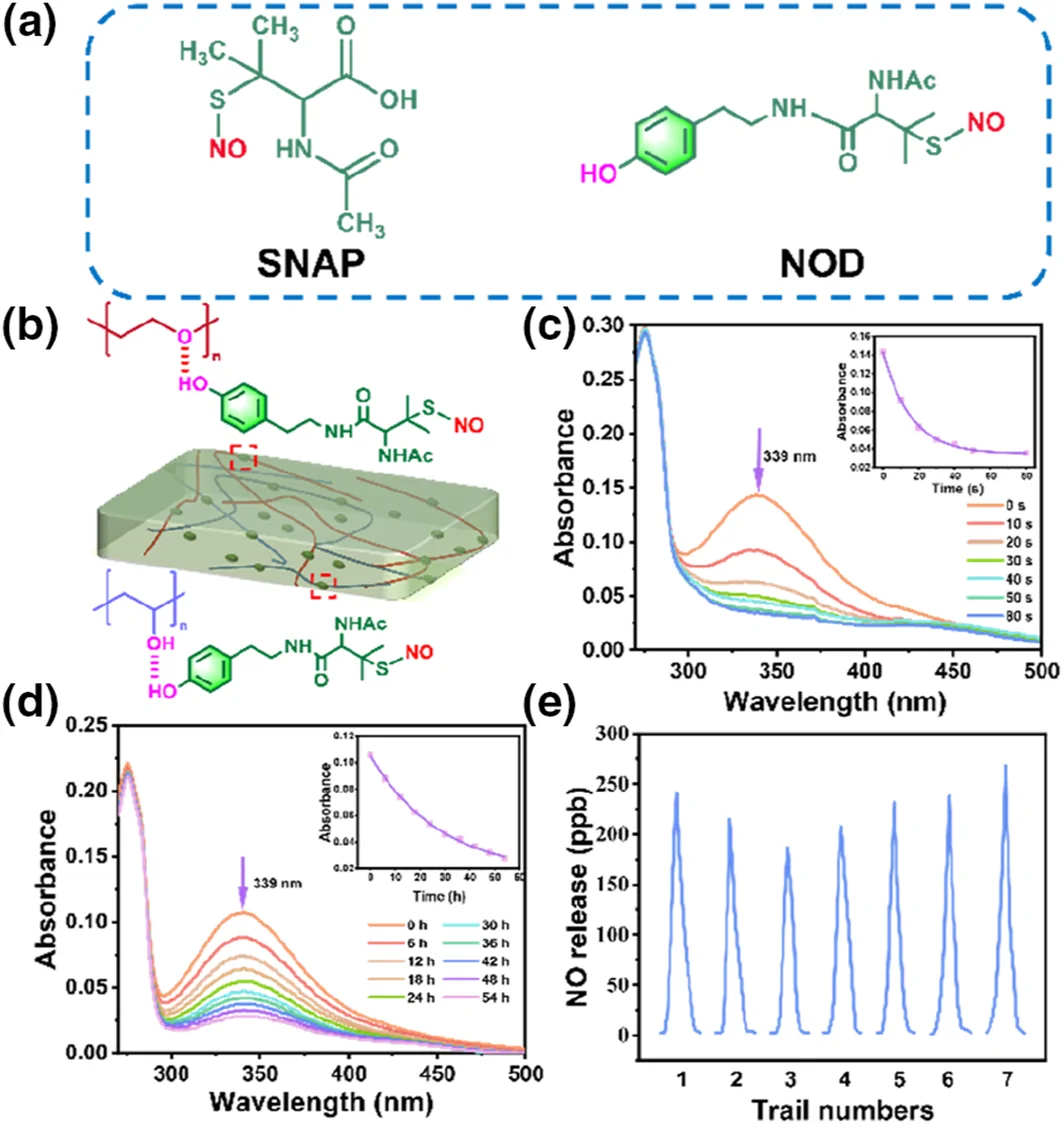
(a) Schematic diagrams of the chemical structures of SNAP and NOD. (b) Schematic illustration of synthetic NOD interacting with the PVA‐PEG hydrogel via hydrogen bonds. (c) UV‐vis spectra of irradiated NOD (150.0 μM) in a mixture of phosphate buffer (pH 7.40, 0.10 M) and MeCN (95:5, v/v) at 25°C. Inset: Plot of absorbance at 339 nm vs. irradiation time. The data were fitted to a first‐order rate equation. (d) UV‐vis spectra of NOD (150.0 μM) in a mixture of phosphate buffer (pH 7.40, 0.10 M) and MeCN (95:5, v/v) at 37°C under dark conditions. Inset: Plot of absorbance at 339 nm vs. time. The data were fitted to a first‐order rate equation. (e) Determination of the amount of NO released (ppb) using chemiluminescence upon photolysis of NOD (0.10 mg, 0.295 μmol) in a mixture of DMSO and deionized water (11 mL, 1:10, v/v) at 25°C.

Hydrogels are characterized by three‐dimensional cross‐linked networks formed via covalent or non‐covalent interactions,^[22]^ and exhibit tunable mechanical stiffness, high swelling capacity, moldability, and excellent biocompatibility.[^23^, ^24^] These properties render them highly suitable for biomedical and tissue engineering applications.[^25^, ^26^, ^27^] Although hydrogels loaded with NO donors such as SNAP or *S*‐nitrosoglutathione (GSNO) have shown broad potential in wound healing, antimicrobial, and anticancer contexts,[^28^, ^29^, ^30^, ^31^] they often also suffer from significant donor leaching.[^32^, ^33^] To address this, we propose a novel strategy involving the design of a NO donor‐hydrogel system incorporating a phenolic hydroxyl group. The latter functionality can form hydrogen bonds with the hydrogel matrix (Figure 1a). This molecular design aims to mitigate leaching, prolong NO release, and enhance release control. We demonstrate that this hydrogen bond‐mediated incorporation strategy offers a robust and versatile platform for achieving sustained and controlled NO release within hydrogel‐based biomedical systems.

## 2 EXPERIMENTAL

### 2.1 Materials

All commercial chemicals were used directly without any purification. Organic solvents, including dichloromethane, methanol, acetonitrile, dimethyl sulfoxide, acetic anhydride, and pyridine, were purchased from Sigma‐Aldrich. Penicillamine (C_5_H_11_NO_2_S) and tyramine (C_8_H_11_NO) were purchased from Macklin. PVA (viscosity = 50–60 mPa·s, degree of hydrolysis >97%) and PEG (average Mn: 2000 g/mol) were purchased from Alladin (Shanghai, China). *E*. *coli* and *S*. *aureus* were purchased from the BeNa Culture Collection (Shanghai, China).

### 2.2 Analytical methods

^1^H/^13^C NMR spectra were obtained using a 400 MHz NMR spectrometer (Bruker Avance). HRMS data were collected using a Shimadzu LCMS‐IT‐TOF liquid chromatography mass spectrometer. All UV‐Vis spectrometric measurements were carried out using a UV‐Vis spectrophotometer (UV‐2600, Shimadzu) with a TCC‐100 temperature controller. Nitric oxide release from NO donor was determined using a NOA analyzer (model 42i, Thermo Scientific).

The functional groups of the hydrogels were investigated by FTIR (T27 Bruker, Germany) in the frequency range 400–4000 cm^−1^. The morphology and microstructure of the hydrogel samples were characterized using SEM (Verios G4 UC, Thermo Scientific, USA). The elemental distribution in the hydrogel samples was assessed by energy‐dispersive X‐ray spectroscopy (EDX). The crystal structures of these samples were examined by X‐ray diffraction (XRD, Smart Lab). The experiment was performed at a voltage of 40 kV and a current of 40 mA over a range of 3°–70° (2θ) at a fixed scan rate of 20°/min. The mechanical properties of the hydrogel were evaluated using a universal testing machine on a dummy shell‐shaped sample measuring 10 mm wide, 50 mm long, and approximately 2 mm thick. The tests were carried out at room temperature at a tensile rate of 100 mm/min.

### 2.3 Synthesis of NO donor

The general route of synthesis of the NO donor (nitric oxide donor (NOD)) is illustrated in Supporting Information S1: Scheme. S1.

*N‐(2,2‐dimethyl‐4‐oxothietan‐3‐yl) acetamide (lactone)*: *D*‐penicillamine (5.001 g, 33.56 mmol) was dissolved in pyridine (15 mL), and acetic anhydride (8.82 mL, 93.96 mmol) was added to the solution. The mixture was stirred at room temperature for 20 h. At the end of the reaction, CHCl_3_ (100 mL) was added, and the reaction mixture was washed with a hydrochloric acid solution (1.0 M, 50 mL × 3), dried with Na_2_SO_4,_ and evaporated under reduced pressure to remove the solvent to give a yellow solid. The yellow solid was washed with petroleum ether to give a pale yellow solid. Finally, the crude product (1.855 g) was obtained after drying under vacuum overnight and was used directly in the next step without further purification. The desired product was characterized by ^1^H NMR (Supporting Information S1: Figure S1). ^1^H NMR (400 MHz, CDCl_3_) *δ* 6.32 (s, 1 H), 5.66 (d, *J* = 7.6 Hz, 1 H), 2.04 (s, 3 H), 1.85 (s, 3 H), 1.62 (s, 3 H).

*2‐Acetamido‐N‐(4‐hydroxyphenethyl)‐3‐mercapto‐3‐me‐thylbutanamide*: The lactone (700.0 mg, 4.05 mmol) was dissolved in CHCl_3_ (30 mL), and tyramine (4.86 mmol, 1.2 equiv.) was added to the solution. The mixture was stirred at room temperature for 24 h and dried. The crude product was purified by column chromatography (MeOH: CH_2_Cl_2_ = 1:35) to give a white powder with a yield of 69.6%. The desired product was characterized by ^1^H/^13^C NMR (Supporting Information S1: Figures S2 and S3) and HRMS. ^1^H NMR (400 MHz, CDCl_3_) *δ* 7.04 (d, *J* = 8.3 Hz, 2H), 6.76 (d, *J* = 8.4 Hz, 2H), 6.56 (d, *J* = 9.7 Hz, 1H), 6.21 (s, 1H), 4.25 (d, *J* = 9.1 Hz, 1H), 3.53 (app. sextet, *J* = 6.2 Hz, 1H), 3.44 (app. sextet, *J* = 6.2 Hz, 1H), 2.74 (t, *J* = 6.9 Hz, 2H), 2.66 (s, 1H), 2.04 (s, 3H), 1.43 (s, 3H), 1.24 (s, 3H). ^13^C NMR (101 MHz, DMSO‐*d*_*6*_) *δ* 169.68, 169.42, 156.08, 129.96, 129.78, 115.50, 61.04, 46.56, 40.89, 34.48, 30.42, 29.59, 22.97. HRMS: m/z (ESI): calculated [M+H]^−^ m/z 311.1430 found 311.1434.

*S‐(3‐acetamido‐4‐((4‐hydroxyphenethyl)amino)‐2‐methyl‐4‐oxobutan‐2‐yl)nitrothioite (NOD)*: Free thiols (200 mg, 0.65 mmol) were dissolved in MeOH (10 mL) and hydrochloric acid (10 mL, 1.0 M). The reaction was kept at 0°C. Concentrated H_2_SO_4_ (2 mL) and an aqueous solution of NaNO_2_ (1.95 mmol, in 2 mL water) were added to the reaction mixture. The reaction was maintained at room temperature for 1 h. The product mixture was extracted with CH_2_Cl_2_ (5 mL × 3), and the organic phase dried using Na_2_SO_4_. After the solvent was removed, the solid product was dried overnight under vacuum in dark conditions to give a green solid with a yield of 69.6%. The desired product was characterized by ^1^H/^13^C NMR (Supporting Information S1: Figures S4 and S5) and HRMS. ^1^H NMR (400 MHz, DMSO‐*d*_*6*_) *δ* 9.17 (s, 1H), 8.49 (t, *J* = 5.4 Hz, 1H), 8.37 (d, *J* = 9.8 Hz, 1H), 6.99 (d, *J* = 8.4 Hz, 2H), 6.65 (d, *J* = 8.5 Hz, 2H), 5.16 (d, *J* = 9.8 Hz, 1H), 3.28 (app septet, *J* = 7.4 Hz, 1H), 3.22 (app septet, *J* = 6.7 Hz, 1H), 2.62–2.58 (m, 2H), 1.91 (s, 3H), 1.88 (s, 3H), 1.84 (s, 3H). ^13^C NMR (101 MHz, DMSO‐*d*_*6*_) *δ* 169.73, 168.50, 156.11, 129.97, 129.67, 115.49, 60.17, 59.39, 40.95, 34.34, 27.17, 25.26, 22.77. HRMS: m/z (ESI): calculated [M+H]^−^ m/z 340.1326 found 340.1319.

### 2.4 UV‐vis spectroscopy of NOD

Kinetic studies of the steady state photolysis and thermal decomposition of NOD (150.0 μM) in a mixture of phosphate buffer (pH 7.40, 0.10 M) and MeCN (95:5, v/v) were carried out using a Shimadzu UV‐visible spectrophotometer UV‐2600 equipped with a TCC‐100 thermostat. NOD was dissolved in a mixture of phosphate buffer (pH 7.40, 0.10 M) and MeCN (95:5, v/v) at 25°C. The corresponding UV‐Vis spectra were recorded after 10 s of irradiation using an external 365 nm UV lamp. The thermal decomposition of NOD was monitored in a mixture of aqueous phosphate buffer (pH 7.40, 0.10 M) and MeCN (95:5, v/v) at 37°C in a dark environment.

The aqueous solubility of NOD was determined under dark conditions at 25°C. Briefly, excess NOD was added to deionized water and continuously shaken until equilibrium was reached. After removal of the undissolved material by centrifugation, the concentration of dissolved NOD in the supernatant was quantified by UV–Vis spectroscopy using the calibration curve shown in Supporting Information S1: Figure S11a. The apparent aqueous solubility of NOD was determined to be 10.27 mg/mL (30.1 mM).

### 2.5 NO release from NOD

The NO gas released from NOD was quantified using a NOA analyzer (model 42i, Thermo Scientific). NOD was dissolved in DMSO (0.1 mg/mL), and 1 mL of this solution was added to a clear quartz reactor containing deionized water (10 mL) at room temperature (25°C). Next, NOD was photolyzed using a 365 nm UV lamp. The experiment was repeated seven times, and the NO‐releasing signal was recorded each time.

### 2.6 Preparation of hydrogels loaded in the absence of and with NOD or SNAP (PVA‐PEG, PVA‐PEG‐NOD‐5, PVA‐PEG‐NOD‐10, PVA‐PEG‐NOD‐15, PVA‐PEG‐SNAP‐10)

A homogeneous solution of PVA (10%, w/v) was obtained by dissolving PVA (10.0 g) in distilled water (90 mL) and stirring at 90°C for 4 h until PVA was completely dissolved. The PVA solution was cooled to room temperature, and PEG was added with mass ratio PEG: PVA = 1:3 to obtain a PVA‐PEG mixture. The PVA‐PEG mixture was evenly divided into five groups, each weighing 1.0 g. To prepare hydrogels with varying NOD donor concentrations, 52.6 mg, 111.1 mg, and 176.5 mg of the NOD donor were added to three of the groups, resulting in hydrogels loaded with 5%, 10%, and 15% (weight percentage, wt%) NOD, respectively (PVA‐PEG‐NOD‐5, PVA‐PEG‐NOD‐10, PVA‐PEG‐NOD‐15). 10 wt% SNAP (114.5 mg, 0.521 mmol) was also added to give PVA‐PEG‐SNAP‐10. (This wt% is equimolar to the 15 wt% NOD hydrogel.) PVA‐PEG and the four samples (5.0 mL) were poured into a mold which was placed in a freezer at −20°C for 20 h, and then warmed to room temperature over 4 h. The freezing‐thawing process was repeated three times for the five hydrogel samples. The hydrogels were lyophilized and stored at 4°C.

### 2.7 Swelling behavior of hydrogels

The swelling behavior of four groups of hydrogels was investigated using the gravimetric method. Firstly, the initial masses (W_0_) of the lyophilized PVA‐PEG, PVA‐PEG‐NOD‐5, PVA‐PEG‐NOD‐10, and PVA‐PEG‐NOD‐15 hydrogels were accurately weighed and recorded. Subsequently, the four groups of samples were immersed in vials containing an appropriate amount of phosphate buffer (pH 7.40). At regular time intervals, the samples were taken out, the residual moisture on the surface was carefully blotted dry, and the sample was weighed and recorded as W_*t*_. Triplicate measurements were performed for each hydrogel composition to ensure statistical reliability. The swelling ratio (SR) was calculated according to the Formula (1):

(1) $$\text{SR}\;\left(\%\right)=\frac{W_{t}\;-\;W_{0}}{W_{0}}\times 100\%$$

### 2.8 NO release properties of hydrogels

Nitric oxide release from the hydrogels was measured using a chemiluminescence NO analyzer (Model 42i, Thermo Scientific, USA). Lyophilized hydrogel specimens with a dry mass of 50.0 ± 1.0 mg were cut into rectangular cuboids measuring 10.0 mm × 10.0 mm × 2.0 mm. The corresponding geometric surface area was 2.8 cm^2^. Each specimen was wrapped in an inert, porous sponge and placed in an amber quartz reactor, which was maintained at 37.0°C ± 0.1°C in a water bath. Phosphate buffer (2 mL, pH 7.40) was added to hydrate the hydrogel and maintain a moist release environment. The entire accessible surface of the specimen was exposed to the hydrated sponge and the surrounding gas phase. Nitrogen was continuously supplied at a mass flow rate of 0.01 kg/h to transport the released NO to the analyzer. Before each set of measurements, the analyzer was calibrated using a zero‐gas reference and a known NO standard under the same gas‐flow and instrumental conditions used for sample analysis. The background signal recorded before sample hydration was subtracted from the measured NO signal. The time‐dependent ppb signal was converted to an NO release rate using the experimentally determined calibration factor, which accounted for the carrier‐gas flow rate and instrumental response. Cumulative NO release was calculated by numerical integration of the baseline‐corrected release rate over the complete release period.

### 2.9 Antimicrobial testing

The antimicrobial activity of the lyophilized PVA‐PEG‐NOD hydrogels was determined by a standard bacteriostatic assay using *E*. *coli* and *S*. *aureus*, respectively. Firstly, the strains stored at −80°C were thawed, transferred to nutrient agar plates, and incubated at 37°C for 24 h. Single colonies were selected and incubated in sterile MRS broth at 37°C for 24 h. The bacterial culture solution (5 mL) was added to a sample (50 mg) of the hydrogel (PVA‐PEG, PVA‐PEG‐NOD‐5, PVA‐PEG‐NOD‐10, or PVA‐PEG‐NOD‐15) in a 12‐well plate. After 24 h of co‐incubation at 37°C, the bacterial suspension (100.0 μL) was taken into the well plate. The dilutions were added to the nutrient agar medium for coating experiments using the 10‐fold dilution method. Three parallel experiments were performed for each set of hydrogel samples. After incubation at 37°C for 24 h, the number of colonies in each group of samples was recorded accurately to verify the antimicrobial performance.

*E*. *coli* and *S*. *aureus* were co‐incubated with PVA‐PEG and PVA‐PEG‐NOD‐15 hydrogels under dark conditions in a constant temperature incubator at 37°C. After 24 h, antimicrobial‐completed bacterial fluids were collected and centrifuged at 5000 rpm for 20 min. The bottom precipitate was washed twice with PBS and centrifuged for 20 min each time. The sample was fixed overnight at 4°C with 2.5% glutaraldehyde. The next day, the bacterial solution was centrifuged for 20 min to remove glutaraldehyde. A series of ethanol/water mixtures (30%, 50%, 70%, 90%, and 100% volume of ethanol, respectively) was left to dehydrate for 15 min, followed by centrifugation for 20 min. The morphology of the *E*. *coli* and *S*. *aureus* was visualized using SEM after treating them with gold at 30 mA for 1 min.

### 2.10 NOD leaching

Each of the lyophilized hydrogels PVA‐PEG‐NOD‐5, PVA‐PEG‐NOD‐10, PVA‐PEG‐NOD‐15, and PVA‐PEG‐SNAP‐10 were cut into 1 cm × 1 cm square slices, weighed, and placed in a mixture of acetonitrile and water (2 mL, 1:9, v/v). The systems were kept in a dark environment at 37°C. The acetonitrile and water mixture was then removed every 12 h or 24 h and replaced with a fresh solvent mixture. The concentrations of NOD and SNAP were calculated using the absorbance at 339 nm and the standard curve. The process was repeated three times for each sample.

### 2.11 Cytotoxicity assay

The cell viability of PVA‐PEG and PVA‐PEG‐NOD‐15 hydrogels was assessed using the Cell Counting Kit‐8 (CCK‐8) assay. Human umbilical vein endothelial cells (HUVEC) were cultured in RPMI‐1640 medium. Cells were seeded in a 96‐well plate at a density of 5 × 10^4^ cells per well and incubated at 37°C. Subsequently, varying concentrations (0.025 mg/mL, 0.05 mg/mL, 0.1 mg/mL, and 0.2 mg/mL) of PVA‐PEG and PVA‐PEG‐NOD‐15 hydrogels were added to each well, followed by a 12 h incubation period. A negative control group, consisting of RPMI‐1640 medium only, was also included. After incubation, the culture medium was removed, and 10 μL of CCK‐8 reagent was added to each well. The plate was then incubated at 37°C until the formation of formazan crystals. The absorbance (OD value) of each well was measured at 450 nm using a Spectrophotometer Multiskan GO (Thermo Scientific, Pittsburgh, PA). The cell viability was calculated using the following Formula (2):

(2) $$\text{Cell}\;\text{Viability}\;\left(\%\right)=\frac{{\text{OD}}_{\text{experimental}}\;-\;{\text{OD}}_{\text{blank}}}{{\text{OD}}_{\text{negative}\;\text{control}}\;-\;{\text{OD}}_{\text{blank}}}$$

### 2.12 Statistical analysis

The corresponding description in the Statistical Analysis section has been revised accordingly. Statistical analyses were performed using GraphPad Prism 9.0 (GraphPad Software). Data are presented as mean ± standard deviation (SD). Comparisons among multiple groups were analyzed using one‐way analysis of variance followed by Tukey's multiple comparison test. Statistical significance was defined as *p* < 0.05, with *p* < 0.05, *p < 0.01, and **p < 0.001 indicating statistically significant differences. In addition, unless otherwise specified, all experiments were performed using three independent replicates (*n* = 3).

## 3 RESULTS AND DISCUSSION

The target SNAP‐modified NO donor (NOD) capable of hydrogen bonding via a phenol was synthesized via a three‐step route (Supporting Information S1: Scheme S1). The overall yield of the final NOD was approximately 18%. The compound was stored at 0°C–5°C. The NO release behavior of NOD in the absence of hydrogel was initially investigated under both photolytic and thermal conditions. Steady‐state photolysis of NOD (150.0 μM) at 365 nm in phosphate buffer‐MeCN (95:5, v/v) at 25°C resulted in a progressive decrease in the characteristic n_O_→π* absorption band of NOD at 339 nm, which eventually plateaued (Figure 1c). Kinetic analysis of the absorbance change at 339 nm showed that the photodecomposition followed a first‐order decay model, yielding an observed rate constant, *k*_obs_ = (6.49 ± 0.25) × 10^−2^ s^−1^ with a half‐life, *t*_1/2_ = 10.7 s. Thermal decomposition studies conducted at 37°C in the same solvent system showed a concentration‐dependent decrease in absorbance at 339 nm (Figure 1d) with first‐order kinetics, giving *k*_obs_ = (3.57 ± 0.15) × 10^−2^ h^−1^ and *t*_1/2_ = 19.4 h. These findings demonstrate the capacity of NOD to release NO in a controllable manner via both photolytic and thermal activation.

The photolytic NO release profile of a solution of NOD was quantitatively monitored using a chemiluminescence‐based NO analyzer, a gold‐standard method known for its high reliability and reproducibility. Upon irradiation with a 365 nm UV lamp, NOD (0.10 mg, 0.295 μmol) in a DMSO/water (1:10, v/v) mixture exhibited an immediate increase in the NO concentration, from a baseline value of ∼2.5 ppb to 200–300 ppb (Figure 1e). The signal remained elevated for 8–10 min until donor depletion, after which it rapidly returned to near baseline. The NO release profile remained highly reproducible over seven consecutive irradiation cycles, indicating reliable and reproducible photoresponsive behavior. Based on the integrated photolysis data, the total NO released was calculated as 767 ± 71 ppb (0.281 μmol), corresponding to an NO release yield of approximately 95%.

**FIGURE 2:**
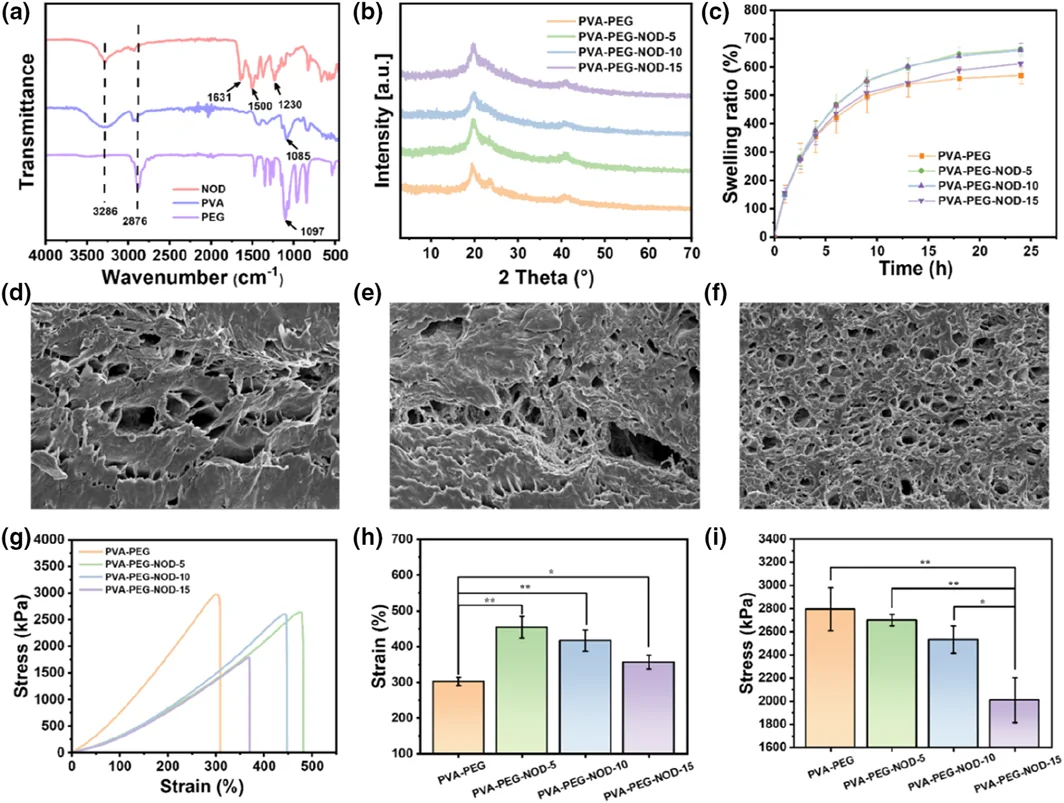
(a) FTIR spectra of PVA‐PEG, PVA‐PEG‐NOD‐5, PVA‐PEG‐NOD‐10, and PVA‐PEG‐NOD‐15 hydrogels. (b) XRD of PVA‐PEG, PVA‐PEG‐NOD‐5, PVA‐PEG‐NOD‐10, and PVA‐PEG‐NOD‐15 hydrogels. (c) Swelling behavior of PVA‐PEG, PVA‐PEG‐NOD‐5, PVA‐PEG‐NOD‐10, and PVA‐PEG‐NOD‐15 hydrogels in PBS. SEM images of (d) PVA‐PEG‐NOD‐5, (e) PVA‐PEG‐NOD‐10, and (f) PVA‐PEG‐NOD‐15 hydrogels. (g) Stress‐strain curves of PVA‐PEG, PVA‐PEG‐NOD‐5, PVA‐PEG‐NOD‐10, and PVA‐PEG‐NOD‐15 hydrogels. (h) Strain of PVA‐PEG, PVA‐PEG‐NOD‐5, PVA‐PEG‐NOD‐10, and PVA‐PEG‐NOD‐15 hydrogels (*n* = 3). (i) Stress of PVA‐PEG, PVA‐PEG‐NOD‐5, PVA‐PEG‐NOD‐10, and PVA‐PEG‐NOD‐15 hydrogels (*n* = 3). *p < 0.05, **p < 0.01.

Three composite hydrogels (PVA‐PEG‐NOD‐5, PVA‐PEG‐NOD‐10, and PVA‐PEG‐NOD‐15) were fabricated by mixing polyvinyl alcohol (PVA), polyethylene glycol (PEG), and NOD, followed by freezing and thawing. The FTIR spectrum of the PVA–PEG hydrogel (Figure 2a) displayed prominent peaks at 2937 cm^−1^ (C–H stretch), 1450 cm^−1^ (C–H bend), 1090 cm^−1^ (C–O–C of PEG), and 3280 cm^−1^ (O–H of PVA), confirming polymer cross‐linking. A O–H/N–H stretch (3500–3100 cm^−1^), C=O stretch (1631 cm^−1^), N–O stretch (1500 cm^−1^), C–N stretch (1230 cm^−1^), and aromatic skeletal vibrations between 1600 and 1400 cm^−1^ were observed for NOD.^[34]^ Critically, NOD‐loaded hydrogels retained donor‐specific peaks at 3280 cm^−1^ (O–H/N–H), 1640 cm^−1^ (C=O), 1505 cm^−1^ (N–O), and 1236 cm^−1^ (C–N), demonstrating successful incorporation of NOD into the hydrogel network.

To provide additional evidence, we performed temperature‐dependent FTIR analysis of the PVA–PEG–NOD and blank PVA–PEG hydrogels under identical conditions. All samples were freeze‐dried before FTIR measurements to minimize the influence of free water on the broad O–H stretching region. The temperature range was limited to 0°C–20°C, which is well below the temperature range associated with potential thermal degradation of the NO donor, ensuring that the observed spectral changes mainly originated from variations in intermolecular interactions rather than donor decomposition.

As shown in Supporting Information S1: Figure S6, the temperature‐dependent FTIR spectra of the PVA–PEG–NOD and blank PVA–PEG hydrogels were recorded to investigate the evolution of hydrogen‐bonding interactions. For the PVA–PEG–NOD hydrogel, the center of the broad O–H stretching band shifted from approximately 3446 cm^−1^ at 0°C to 3467 cm^−1^ at 10°C and 3473 cm^−1^ at 20°C, corresponding to an overall blue shift of approximately 27 cm^−1^. In comparison, the blank PVA–PEG hydrogel exhibited only a minor shift from approximately 3448 to 3452 cm^−1^ over the same temperature range. Because heating weakens hydrogen‐bonded associations and promotes the transition of associated O–H groups toward less‐associated hydroxyl states, temperature‐induced blue shifts of the O–H stretching band are commonly used as evidence for hydrogen‐bond rearrangement in polymer systems.[^35^, ^36^, ^37^] Previous temperature‐dependent FTIR studies have demonstrated that the weakening and breaking of hydrogen bonds upon heating are accompanied by shifts of O–H stretching vibrations toward higher wavenumbers, providing insight into the evolution of the hydrogen‐bond network.^[38]^ Similarly, hydrogen bonding among hydroxyl‐containing polymer chains is known to influence the local chemical environment and network structure of PVA/PEG‐based hydrogels.^[39]^ Therefore, the substantially larger temperature‐dependent shift observed in the NOD‐containing hydrogel suggests the presence of additional temperature‐responsive hydrogen‐bonding interactions involving the phenolic hydroxyl group of NOD. These results support the involvement of NOD in the hydrogen‐bonding network of the PVA/PEG matrix, providing further evidence for the proposed donor‐retention mechanism.

The crystallinity of PVA, PEG, NOD, and their composite hydrogels was analyzed by XRD (Figure 2b). Supporting Information S1: Figure S7 gives the XRD of PVA, PEG, and NOD for comparison. NOD displayed multiple sharp peaks (2*θ* = 9.2°, 16.6°, 19.8°, 23.2°), confirming its crystalline structure. In the PVA‐PEG hydrogel, the characteristic PEG peak at 23.4° demonstrated the successful incorporation of PEG into the matrix. The XRD patterns of NOD‐loaded hydrogels (Figure 2a) closely resembled that of the PVA‐PEG control, showing only peaks associated with PVA and PEG (e.g., 19.6°, 23.1°, and 41.2°). No diffraction peaks corresponding to NOD were detected in any hydrogel formulation, likely due to its low loading amount and high dispersion within the polymer network. Importantly, these results demonstrate that the incorporation of NOD does not alter the crystalline structure of the PVA‐PEG composite hydrogel.

The swelling behavior of hydrogels, which critically influences their drug release kinetics, was evaluated gravimetrically. As shown in Figure 2c, all hydrogel groups exhibited a rapid increase in mass within the first 9 h, followed by a more gradual increase in mass to reach the swelling equilibrium value within 24 h. Incorporation of the NOD into the hydrogel significantly enhanced the swelling capacity; however, higher NOD loadings resulted in a progressive reduction in the SR. Specifically, the PVA‐PEG hydrogel (without NOD) showed a SR of 570%, while PVA‐PEG‐NOD‐5, PVA‐PEG‐NOD‐10, and PVA‐PEG‐NOD‐15 were 662%, 660%, and 612%, respectively. This non‐monotonic trend can be explained by competing effects; hydrogen bonding introduced by NOD enhances hydrophilicity and initial swelling, but excessive crosslinking at higher donor content restricts polymer chain mobility and water penetration, thereby limiting further swelling.^[40]^ These findings highlight the role of donor‐mediated hydrogen bonding in modulating both the structure and transport properties of NO‐releasing hydrogels.

The morphological and structural features of PVA‐PEG and NOD‐loaded PVA‐PEG hydrogels were investigated using SEM microscopy. All lyophilized hydrogels exhibited an interconnected porous architecture.^[41]^ The control PVA‐PEG hydrogel (Supporting Information S1: Figure S8) displayed large, irregular pores ranging from 10 to 40 μm. In contrast, NOD‐incorporated hydrogels (Figure 2d–f) showed more densely packed and uniformly distributed pores with diameters between 2 and 25 μm. As the NOD content increased, the pore structure became progressively finer and more honeycomb‐like, indicating enhanced cross‐linking density likely arising from hydrogen bonding between the hydroxyl groups of NOD and polymer chains. This microstructural evolution, from coarse and heterogeneous to compact and uniform, demonstrates the role of NOD in modulating hydrogel morphology. EDS spectroscopy results further confirmed the uniform incorporation of NOD, with sulfur and nitrogen signals present only in NOD‐loaded hydrogels (Supporting Information S1: Figure S9b–d), in contrast to the carbon and oxygen composition of the control sample (Supporting Information S1: Figure S9a).

To enable a more rigorous comparison between NOD‐ and SNAP‐loaded hydrogels, the donor loading parameters and NO release performance were normalized based on donor molar amounts rather than donor mass percentages alone. As summarized in Supporting Information S1: Table S1, the hydrogel mass, donor weight percentage, donor molar amount, theoretical NO content, actual donor loading, cumulative NO release, and NO recovery were compared for PVA–PEG–NOD‐15 and PVA–PEG–SNAP‐10. Despite comparable theoretical NO loading, PVA–PEG–NOD‐15 exhibited a higher NO recovery (6.45%) than PVA–PEG–SNAP‐10 (3.92%). This result further supports that the introduction of the phenolic hydroxyl group improves donor retention within the PVA/PEG network and enables more sustained NO release compared with the conventional SNAP‐loaded hydrogel.

Based on the normalized leaching analysis, PVA–PEG–NOD‐15 exhibited a gradual donor release profile, with approximately 53.4%, 74.5%, and 99.9% of the loaded donor released after 12 h, 3 days, and 13 days, respectively. In comparison, PVA–PEG–SNAP‐10 exhibited a significantly faster release behavior, achieving approximately 93.1% donor release within 5 days and nearly complete donor release within 7 days. More importantly, the SNAP donor signal decreased rapidly during the initial release period, whereas the characteristic NOD signal remained detectable throughout the 13‐day monitoring period. Regarding UV–Vis quantification, the absorbance at 339 nm was selected based on the characteristic absorption of intact nitrosothiol donors. Calibration curves were established using intact NOD and SNAP standards, and the donor concentration was calculated accordingly. Because the decomposition products made a negligible contribution at this wavelength under the experimental conditions, the 339 nm signal primarily reflects intact donor molecules. Therefore, the measured values represent the apparent leaching behavior of intact NO donors from the hydrogel matrix.

In freeze–thawed PVA hydrogels, PVA crystalline domains and hydrogen‐bonded chain aggregates act as physical cross‐linking points, and their abundance and organization strongly affect tensile strength, modulus, and extensibility. Previous studies have shown that increased crystallinity and physical cross‐link density generally enhance the mechanical strength of PVA‐based hydrogels, whereas changes in chain packing and network structure can markedly alter their tensile behavior.^[42]^ Accordingly, at higher NOD loading, the small NOD molecules may partially interfere with PVA chain association and the formation of crystalline physical cross‐links, thereby decreasing the effective cross‐link density and tensile strength.^[43]^ In addition, NOD may exert a mild molecular plasticizing effect by increasing polymer‐chain mobility and free volume. Such plasticization is generally associated with enhanced deformability and elongation but reduced stiffness or tensile resistance.^[44]^ Changes in pore morphology may also contribute to the observed mechanical behavior because larger or less uniform pores can introduce local stress‐concentration sites.^[45]^ The corresponding discussion has been revised accordingly.

The mechanical and tensile properties of the hydrogels were systematically evaluated, revealing their robust yet tunable nature. While pure PVA‐PEG hydrogel exhibited high tensile strength (nearly 3000 kPa at 300% elongation), the incorporation of NOD significantly altered these properties. With increasing NOD content, the tensile strength progressively decreased (2700 kPa for 5%, 2532 kPa for 10%, and 2008 kPa for 15% NOD), while elongation initially increased to 454% at 5% NOD before declining to 356% at 15% loading (Figure 2g–i). This mechanical behavior reflects competing effects; the abundant polar groups (−OH) in NOD provide additional hydrogen bonding sites that enhance chain mobility and initial elongation, whereas excessive NOD incorporation disrupts the optimal PVA‐PEG cross‐linking network, reducing overall strength and ultimately limiting extensibility. Importantly, all formulations maintained excellent mechanical properties (strength >1500 kPa, elongation >300%), attributable to the dense hydrogen‐bonded network formed during freeze‐thaw cycling.^[31]^

**FIGURE 3:**
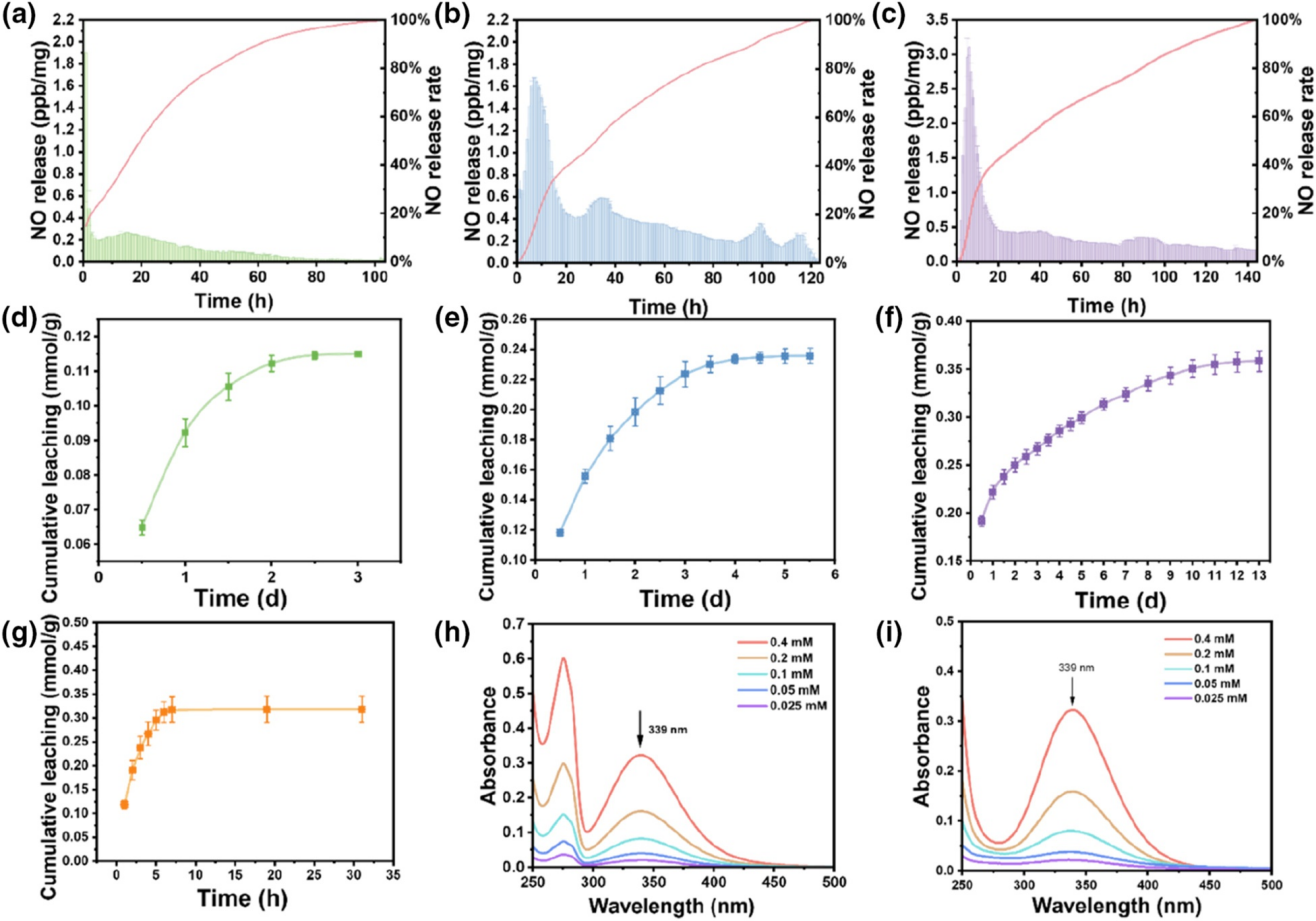
In vitro NO release profile of (a) PVA‐PEG‐NOD‐5, (b) PVA‐PEG‐NOD‐10, (c) PVA‐PEG‐NOD‐15. Cumulative NO release of NOD molecules from (d) PVA‐PEG‐NOD‐5, (e) PVA‐PEG‐NOD‐10, (f) PVA‐PEG‐NOD‐15, and of SNAP molecules from (g) PVA‐PEG‐SNAP‐10 hydrogels. (h) Plot of absorbance vs. wavelength for NOD. (i) Plot of absorbance versus wavelength for SNAP.

The NO release profiles of the NOD‐loaded hydrogels were quantitatively assessed in vitro using chemiluminescence detection of NO at 37°C. As illustrated in Figure 3a–c and Supporting Information S1: Figure S10, the PVA‐PEG‐NOD hydrogels exhibited sustained NO release compared with PVA‐PEG‐SNAP (∼7 h), with both the cumulative release amount and duration increasing proportionally with NOD loading.

The PVA‐PEG‐NOD‐5 hydrogel released a maximum NO burst concentration of 1.9 ppb/mg and continued NO release for 103 h (Figure 3a). In PVA‐PEG‐NOD‐10 and PVA‐PEG‐NOD‐15, the release durations were even longer (120 and 144 h, respectively), with the latter achieving a maximum NO concentration of 3.10 ppb/mg (Figure 3b,c). In contrast, the SNAP‐loaded hydrogel (PVA‐PEG‐SNAP‐10, prepared at the same molar loading as PVA‐PEG‐NOD‐15) showed a higher initial NO burst (10.63 ppb/mg) but a significantly shorter release period of only 10 h (Supporting Information S1: Figure S10). Cumulative NO release totals (Figure 3d–g) were 13.1 ppb/mg (PVA‐PEG‐NOD‐5), 44.61 ppb/mg (PVA‐PEG‐NOD‐10), 63.25 ppb/mg (PVA‐PEG‐NOD‐15), and 33.98 ppb/mg (PVA‐PEG‐SNAP‐10). Notably, after 10 h, the hydrogels incorporating NOD had released only 24%–32% of their total NO, whereas the SNAP hydrogel was fully depleted within this timeframe. This prolonged and stabilized release is attributed to the hydrogen‐bonding interactions between the polar functional groups (–OH) in NOD and the PVA‐PEG matrix. In contrast, the absence of hydrogen bonding interactions in the SNAP‐loaded system is proposed to result in the rapid NO donor leakage and the shorter duration of the NO release. These findings underscore the efficacy of hydrogen‐bonded donor incorporation in achieving sustained NO release, highlighting the potential of NOD‐functionalized hydrogels for long‐term biomedical applications.

**FIGURE 4:**
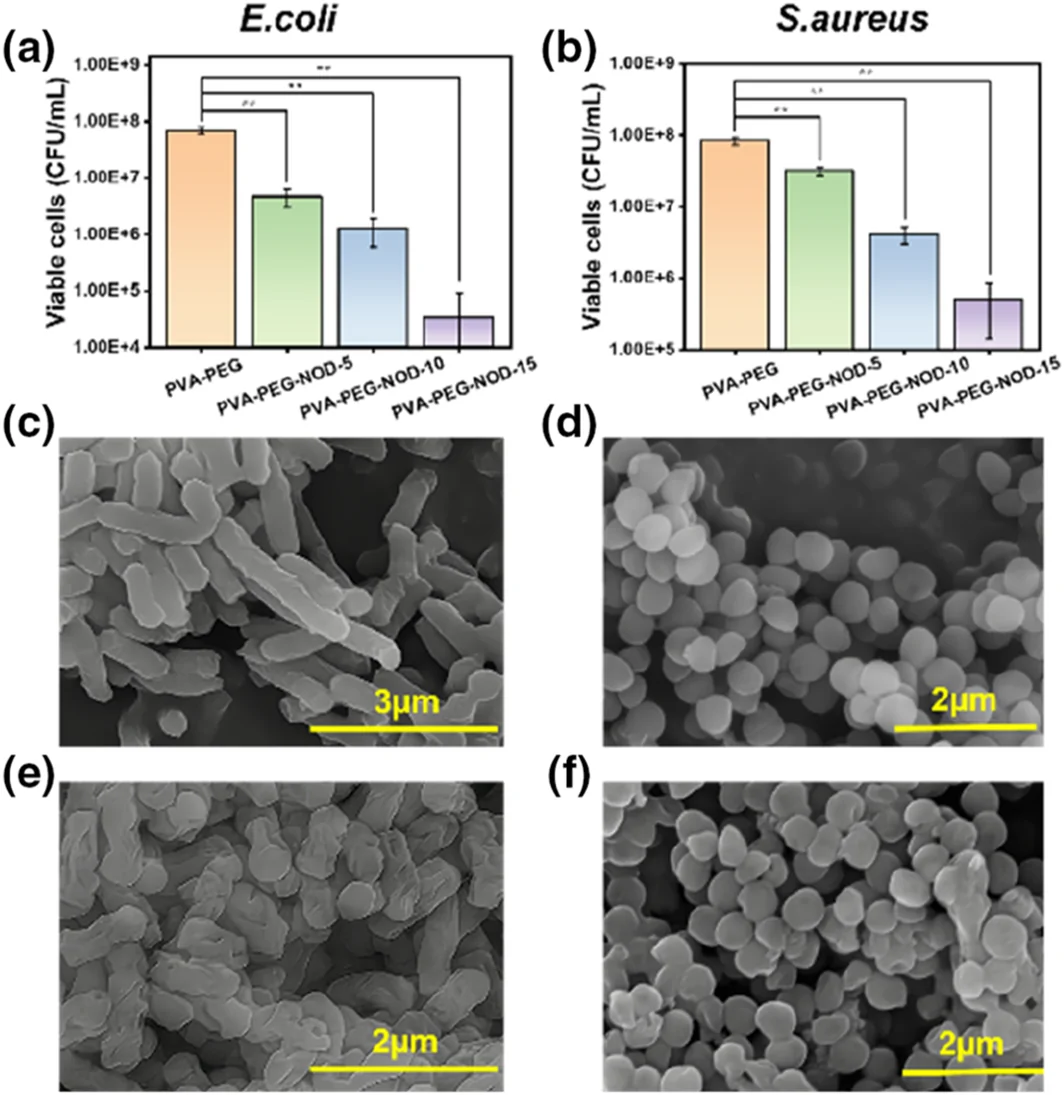
The antibacterial performance of various hydrogels against (a) *Escherichia coli* and (b) *Staphylococcus aureus* over 24 h (*n* = 3). **p < 0.01. (c, e) are SEM images of *E*. *coli* co‐cultured with PVA‐PEG and PVA‐PEG‐NOD‐15 hydrogels, respectively. (d, f) are SEM images of *S*. *aureus* co‐cultured with PVA‐PEG and PVA‐PEG‐NOD‐15 hydrogels, respectively.

Nitric oxide plays a pivotal role in innate immunity by exerting antimicrobial activity through multiple mechanisms.^[46]^ The antibacterial efficacy of these NOD‐hydrogels was therefore evaluated against *E*. *coli* (Figure 4a) and *S*. *aureus* (Figure 4b) using the PVA‐PEG hydrogel as a negative control. While the control showed no inherent antibacterial activity, NOD‐loaded hydrogels exhibited dose‐dependent antimicrobial effects. For *S*. *aureus*, bacterial counts were reduced by 0.4, 1.3, and 2.2 log units for the 5%, 10%, and 15% NOD loaded hydrogels, corresponding to inhibition rates of ∼62%, 96%, and 99%, respectively. For *E*. *coli*, the reductions were 1.2, 1.7, and 3.31 log units with efficiencies of ∼93%, 98%, and >99.9%, indicating even stronger activity against this Gram‐negative bacterium. Scanning electron microscopy (SEM) further revealed that bacteria treated with PVA‐PEG‐NOD‐15 hydrogels displayed wrinkled and ruptured membranes (Figure 4e,f), in contrast to the intact morphology of the controls (Figure 4c,d). This membrane disruption likely leads to cytoplasmic leakage and impaired biofilm formation, underscoring the potential of NOD‐loaded hydrogels as effective, long‐lasting antibacterial wound dressings.

To verify that the antibacterial effect was primarily mediated by NO release, spent PVA–PEG–NOD hydrogels after NO depletion were further evaluated. As shown in Supporting Information S1: Figure S12, the Spent NOD hydrogel produced viable bacterial counts comparable to those of the blank PVA–PEG hydrogel for both *E*. *coli* and *S*. *aureus*, whereas PVA–PEG–NOD‐15 markedly reduced bacterial viability. This result indicates that depletion of releasable NO largely eliminated the antibacterial effect and that the PVA–PEG matrix itself contributed minimally to bacterial killing. Soluble NOD at an equivalent donor concentration also significantly decreased the viable counts of both strains, further confirming that the antibacterial activity originated from NOD‐derived NO. Notably, PVA–PEG–NOD‐15 exhibited greater antibacterial efficacy than soluble NOD, likely due to the hydrogel network's ability to enable controlled and sustained NO release, thereby prolonging bacterial exposure to an effective NO concentration. These results confirm that the antibacterial activity primarily arises from NOD‐derived NO rather than the PVA–PEG matrix.

Based on its sustained NO release profile and superior antibacterial performance, the PVA‐PEG‐NOD‐15 hydrogel was selected for cytocompatibility assessment. As shown in Supporting Information S1: Figure S13, both PVA‐PEG and PVA‐PEG‐NOD‐15 hydrogels maintained cell viability in HUVEC above 100% at concentrations of 0.025, 0.05, and 0.1 mg/mL, indicating no significant cytotoxicity within this range. Even at an elevated concentration of 0.2 mg/mL, HUVEC viability remained above 85%, confirming minimal adverse effects under the tested conditions. Notably, a non‐monotonic trend in cell viability was observed with increasing NOD content, suggesting that low concentrations of released NO promote cellular proliferation, whereas higher levels gradually exert inhibitory effects. These results collectively demonstrate the favorable cytocompatibility of the PVA‐PEG‐NOD hydrogel system, supporting its potential suitability for biomedical applications. As shown in Supporting Information S1: Figure S14, both PVA–PEG and PVA–PEG–NOD‐15 maintained fibroblast viability above approximately 80% over the tested concentration range of 0.025–0.2 mgm L^−1^ after 48 h of exposure, indicating acceptable short‐term cytocompatibility. These findings are consistent with previous reports showing favorable cytocompatibility of PVA‐ and PEG‐based hydrogel systems toward fibroblasts and their suitability for wound‐dressing applications. The 48 h exposure period was selected to reflect the intended use of the present NO‐releasing hydrogel as a replaceable wound dressing. Because continuous NO delivery depends on the remaining donor content and the local wound condition, the dressing is intended to be periodically replaced rather than used as a permanent implant.

## 4 CONCLUSION

In summary, this work presents a hydrogen bond‐integrated NO donor (NOD) that enables sustained and controllable NO release within PVA‐PEG hydrogels by leveraging specific hydrogen‐bonding molecular interaction with the polymer network. The NOD molecule demonstrated well‐defined photolytic (*t*_1/2_ = 10.7 s) and thermal (*t*_1/2_ = 19.4 h) decomposition behavior. FTIR and SEM analyses verified successful NO donor incorporation and microstructural densification of the hydrogel matrix. While NOD addition improved material ductility (strain >300%), it moderately reduced tensile strength. The hydrogen bonding effect significantly reduced NO donor leaching, allowing extended NO release over 144 h markedly longer than conventional SNAP‐doped equivalents. The resulting hydrogels exhibited strong broad‐spectrum antibacterial efficacy (up to 99% against *E*. *coli* and *S*. *aureus*), antibiofilm capability, and high cytocompatibility. This hydrogen‐bonded donor approach provides a highly promising platform for developing durable NO‐releasing biomaterials and a conceptual framework for managing burst release in therapeutic systems via hydrogen bonding. Subsequent studies will focus on in vivo validation using wound infection models and exploration toward other implantable device applications.

## CONFLICT OF INTEREST STATEMENT

The authors declare no conflicts of interest.

## ETHICS STATEMENT

This study did not involve human participants or animal experiments. All cell‐based experiments were performed using established cell lines. Therefore, ethical approval and informed consent were not required.

## Supporting information

Supporting Information S1

## Data Availability

The data that support the findings of this study are available from the corresponding author upon reasonable request.

## References

[1] M. Carlström , Nat. Rev. Nephrol. 2021, 17, 575. 34075241 10.1038/s41581-021-00429-z PMC8169406

[2] J. Shao , H. Jing , P. Wei , X. Fu , L. Pang , Y. Song , K. Ye , M. Li , L. Jiang , J. Ma , R. Li , R. Si , Z. Peng , G. Wang , J. Xiao , Nat. Energy 2023, 8, 1273.

[3] H. Liao , S. Hu , H. Yang , L. Wang , S. Tanaka , I. Takigawa , W. Li , H. Fan , J. P. Gong , Nature 2025, 644, 89. 40770436 10.1038/s41586-025-09269-4 PMC12328221

[4] J. Zeng , X. A. Zhao , Z. Liang , I. Hidalgo , M. Gebert , P. Fan , C. Wenzl , S. G. Gornik , J. U. Lohmann , Nat. Commun. 2023, 14, 8001. 38049411 10.1038/s41467-023-43705-1 PMC10696095

[5] C. Bogdan , Nat. Immunol. 2001, 2, 907. 11577346 10.1038/ni1001-907

[6] G. Zhao , Y.‐Y. Guo , S. Yao , X. Shi , L. Lv , Y.‐L. Du , Nat. Commun. 2020, 11, 1614. 32235841 10.1038/s41467-020-15420-8 PMC7109123

[7] S. M. Andrabi , N. S. Sharma , A. Karan , S. M. S. Shahriar , B. Cordon , B. Ma , J. W. Xie , Adv. Sci. 2023, 10, 38. 10.1002/advs.202303259 PMC10602574 37632708

[8] L. Chang , J. Li , J. Zhang , F. Yuan , L.‐L. Gao , J.‐W. Shen , J. Zhang , Y. Guo , Chin. Chem. Lett. 2025, 37, 111745.

[9] W. Jiang , Z. Guo , Q. Wang , Z. Chen , W. Dong , Q. Liang , Y. Hao , H. Pan , C. Zeng , H. Liu , Y. Wang , Nat. Biomed. Eng. 2025, 9, 1486. 40316687 10.1038/s41551-025-01385-w

[10] H. Kim , Y. R. Lee , H. Jeong , J. Lee , X. Wu , H. Li , J. Yoon , Smart Mol. 2023, 1, e20220010. 40625653 10.1002/smo.20220010 PMC12118215

[11] C. He , J. Wang , L. Fu , C. Zhao , J. Huo , Chin. Chem. Lett. 2022, 33, 1051.

[12] S. T. Emmerling , J. Maschita , B. V. Lotsch , JACS 2023, 145, 7800. 10.1021/jacs.2c11967 PMC10103124 36976754

[13] A. C. Midgley , Y. Z. Wei , Z. J. Li , D. L. Kong , Q. Zhao , Adv. Mater. 2020, 32, 13. 10.1002/adma.201805818 31423672

[14] S. M. Andrabi , N. S. Sharma , A. Karan , S. M. S. Shahriar , B. Cordon , B. Ma , J. Xie , Adv. Sci. 2023, 10, e2303259. 10.1002/advs.202303259 PMC10602574 37632708

[15] J. O. Lundberg , E. Weitzberg , Cell 2022, 185, 2853. 35931019 10.1016/j.cell.2022.06.010

[16] M. D. Pagel , Nat. Mater. 2025, 24, 16. 39753857 10.1038/s41563-024-02089-3

[17] C. Ye , X. Zhou , Y. Zhang , Y. Wang , J. Wang , C. Zhang , R. Woodward‐Massey , C. Cantrell , R. L. Mauldin , T. Campos , R. S. Hornbrook , J. Ortega , E. C. Apel , J. Haggerty , S. Hall , K. Ullmann , A. Weinheimer , J. Stutz , T. Karl , J. N. Smith , A. Guenther , S. Song , Nat. Commun. 2023, 14, 7995. 38042847 10.1038/s41467-023-43866-z PMC10693570

[18] Z. Hu , H. J. C. T. Wessels , T. van Alen , M. S. M. Jetten , B. Kartal , Nat. Commun. 2019, 10, 1244. 30886150 10.1038/s41467-019-09268-w PMC6423088

[19] T. Yang , A. N. Zelikin , R. Chandrawati , Adv. Sci. 2018, 5, 20. 10.1002/advs.201701043 PMC6010811 29938181

[20] Y. Zhou , J. Y. Tan , J. F. Wu , Q. Zhang , J. Andre , C. W. Xi , Z. Chen , M. E. Meyerhoff , Acta Biomater. 2019, 90, 112. 30980938 10.1016/j.actbio.2019.04.021 PMC6513704

[21] M. J. Goudie , J. Pant , H. Handa , Sci. Rep. 2017, 7, 13. 29051609 10.1038/s41598-017-14012-9 PMC5648791

[22] X. Zhang , D. Li , X. Yang , L. Wang , G. Li , T.‐W. Wong , T. Li , W. Yang , Z. Luo , Sci. (New York, N.Y.) 2025, 387, 967. 10.1126/science.adq2711 40014727

[23] G. Zhang , X. Fu , D. Zhou , R. Hu , A. Qin , B. Z. Tang , Smart Mol. 2023, 1, e20220008. 40625652 10.1002/smo.20220008 PMC12118199

[24] J. Ding , T. Wang , Z. Lin , Z. Li , J. Yang , F. Li , Y. Rong , X. Chen , C. He , Nat. Commun. 2025, 16, 1222. 39890820 10.1038/s41467-025-56137-w PMC11785995

[25] T.‐C. Ho , C.‐C. Chang , H.‐P. Chan , T.‐W. Chung , C.‐W. Shu , K.‐P. Chuang , T.‐H. Duh , M.‐H. Yang , Y.‐C. Tyan , Molecules 2022, 27, 2902. 35566251 10.3390/molecules27092902 PMC9104731

[26] M. Kulbay , K. Y. Wu , D. Truong , S. D. Tran , Smart Mol. 2024, 2, e20230021. 40625521 10.1002/smo.20230021 PMC12118312

[27] C. He , J. Wang , L. Fu , W. Wei , Chin. Chem. Lett. 2024, 35, 109037.

[28] A. Karimi , M. Navidbakhsh , Mater. Technol. 2013, 29, 90.

[29] J. R. Chen , X. T. Shi , L. Ren , Y. J. Wang , Carbon 2017, 111, 18.

[30] G. Li , D. H. Zhang , S. H. Qin , Nanomaterials 2019, 9, 15.

[31] X. L. Xiao , G. Z. Wu , H. T. Zhou , K. Qian , J. L. Hu , Polymers 2017, 9, 15.30970691

[32] A. A. Zahid , R. Ahmed , S. Raza ur Rehman , R. Augustine , M. Tariq , A. Hasan , Int. J. Biol. Macromol. 2019, 136, 901. 31229545 10.1016/j.ijbiomac.2019.06.136

[33] J. Kim , D. M. Francis , L. F. Sestito , P. A. Archer , M. P. Manspeaker , M. J. O’Melia , S. N. Thomas , Nat. Commun. 2022, 13, 1479. 35304456 10.1038/s41467-022-29121-x PMC8933465

[34] Y. Qian , M. K. Chug , H. Massoumi , E. J. Brisbois , Mater. Adv. 2022, 3, 6270.

[35] D. Li , J. Brisson , Polymer 1998, 39, 801.

[36] A. Gomez‐Lopez , N. Ayensa , B. Grignard , L. Irusta , I. i. Calvo , A. J. Müller , C. Detrembleur , H. Sardon , ACS Polym. Au 2022, 2, 194. 35698472 10.1021/acspolymersau.1c00053 PMC9185748

[37] M. Ferreras Moreno , C. J. Stinson , R. G. Shepherd , I. D. Welsh , C. Altaner , D. L. Crittenden , J. Phys. Chem. B 2020, 124, 4924. 32441522 10.1021/acs.jpcb.0c02793

[38] Y. Mikhaylova , G. Adam , L. Häussler , K. J. Eichhorn , B. Voit , J. Mol. Struct. 2006, 788, 80.

[39] H. S. Mansur , R. L. Oréfice , A. A. P. Mansur , Polymer 2004, 45, 7193.

[40] S. Khan , N. M. Ranjha , Polym. Bull. 2014, 71, 2133.

[41] Z. S. Ye , H. L. Lu , G. Q. Chai , C. L. Wu , J. Chen , L. F. Lv , Polym. Int. 2023, 72, 27.

[42] S. Gupta , S. Goswami , A. Sinha , Biomed. Mater. 2012, 7, 015006. 22287550 10.1088/1748-6041/7/1/015006

[43] K. Ou , X. Dong , C. Qin , X. Ji , J. He , Mater. Sci. Eng. C 2017, 77, 1017. 10.1016/j.msec.2017.03.287 28531973

[44] T. Yang , J. Wu , Y. Yao , K. Wang , Q. Zhang , Q. Fu , Polymer 2024, 301, 127031.

[45] S. Bose , M. Akbarzadeh Khorshidi , C. Lally , J. Mech. Behav. Biomed. Mater. 2025, 161, 106787. 39549471 10.1016/j.jmbbm.2024.106787

[46] G. Zheng , R. Li , P. Wu , L. Zhang , Y. Qin , S. Wan , J. Pei , P. Yu , K. Fu , M. E. Meyerhoff , Y. Liu , Y. Zhou , Int. J. Biol. Macromol. 2023, 252, 126371. 37595726 10.1016/j.ijbiomac.2023.126371

