# Supplementary material for: Hydrogen‐bond‐mediated retention of a phenolic SNAP analogue in polyvinyl alcohol/PEG hydrogels for sustained nitric oxide release and antibacterial activity
Source: Smart Mol. 2026 Aug 31:e70099. Online ahead of print. doi: 10.1002/smo2.70099 (PMC13530438; doi:10.1002/smo2.70099)
Supplement: Supplementary file 1 [file SMO2-9999-0-s001.docx]

**Supporting Information**

**Hydrogen-Bond-Mediated Retention of a Phenolic SNAP Analogue in PVA/PEG Hydrogels for Sustained Nitric Oxide Release and Antibacterial Activity**

Yixin Zhu^a,#^, Peixuan Wu^a,#^, Shuting Zhang^b^, Congjun Xu^b^, Guanghua Xia^a^,

Jiangli Fan^c^, Nicola E. Brasch^d^, Yang Zhou^a^, Yuanyuan Liu^a,*^

^a^ Key Laboratory of Advanced Materials of Tropical Island Resources of Ministry of Education and School of Chemistry and Chemical Engineering, Hainan University, Haikou 570228, China

*^b^ Key Laboratory of Tropical Biological Resources of Ministry of Education and One Health Institute, School of Pharmaceutical Sciences, Hainan University, Haikou 570228, China*

*^c^ State Key Laboratory of Fine Chemicals, Dalian University of Technology, 2 Linggong Road, Dalian 116024, China*

*^d^School of Science, Auckland University of Technology, Private Bag 92006, Auckland 1142, New Zealand*

*^#^ These authors contributed equally to this work.*

Scheme. S1. Synthesis of NO donor.

Fig. S1. ^1^H NMR spectrum of *N*-(2,2-dimethyl-4-oxothietan-3-yl)acetamide.

Fig. S2. ^1^H NMR spectrum of 2-acetamido-*N*-(4-hydroxyphenethyl)-3-mercapto-3-methylbutanamide.

Fig. S3. ^13^C NMR spectrum of 2-acetamido-*N*-(4-hydroxyphenethyl)-3-mercapto-3-methylbutanamide.

Fig. S4. ^1^H NMR spectrum of NOD.

Fig. S5. ^13^C NMR spectrum of NOD.

Fig. S6. Temperature-dependent FTIR spectra of PVA–PEG–NOD-10 and PVA–PEG hydrogels measured at 0, 10, and 20 °C.

Fig. S7. XRD of PVA, PEG, and NOD.

Fig. S8. SEM images of PVA-PEG hydrogels.

Fig. S9. EDS analysis of (a) PVA-PEG, (b) PVA-PEG-NOD-5, (c) PVA-PEG-NOD-10, and (d) PVA-PEG-NOD-15.

Fig. S10. In vitro NO release profile of PVA-PEG-SNAP-10 hydrogels.

Fig. S11. Standard calibration curves for (a) NOD and (b) SNAP.

Table S1. Normalized comparison of donor loading and NO release performance between PVA–PEG–NOD-15 and PVA–PEG–SNAP-10 hydrogels.

| Sample | PVA–PEG–NOD-15 | PVA–PEG–SNAP-10 |
| --- | --- | --- |
| Hydrogel mass | 50 mg | 50 mg |
| Donor wt% | 15% | 10% |
| Donor amount | 0.44mmol/g | 0.45mmol/g |
| Theoretical NO content | 13.27mg/g | 13.64mg/g |
| Actual donor loading | 0.359 mmol/g （12.2%） | 0.318mmol/g （7.0%） |
| Cumulative NO release | 23.17 μmol g⁻¹ hydrogel | 12.45 μmol g⁻¹ hydrogel |
| NO recovery | 6.45% | 3.92% |

Fig. S12. Antibacterial activities of PVA–PEG hydrogel (I), Spent NOD hydrogel (II), Soluble NOD at an equivalent donor concentration (III), and PVA–PEG–NOD-15 hydrogel (IV) against (a) *E. coli* and (b) *S. aureus* after 24 h of incubation (n = 3). Data are presented as mean ± SD. **P < 0.01.

Fig. S13. Cytotoxicity of PVA-PEG and PVA-PEG-NOD-15 hydrogels in human umbilical vein endothelial cells (HUVEC).

Fig. S14. Cytotoxicity of PVA–PEG and PVA–PEG–NOD-15 hydrogels against fibroblasts after 48 h of incubation.
